# The Effect and Mechanism of Chinese Herbal Formula Sini Tang in Heart Failure after Myocardial Infarction in Rats

**DOI:** 10.1155/2018/5629342

**Published:** 2018-06-27

**Authors:** Yuhan Zhu, Jing Zhao, Qingqing Han, Zhen Wang, Zhaobo Wang, Xin Dong, Jiebai Li, Lei Liu, Xiaoxu Shen

**Affiliations:** ^1^Dongzhimen Hospital Affiliated to Beijing University of Chinese Medicine, Beijing 100700, China; ^2^Cardiology Department, Dongzhimen Hospital Affiliated to Beijing University of Chinese Medicine, Beijing 100700, China; ^3^Lab for Research on Theory of Qi-Blood of Integrative Medicine, Beijing 100700, China

## Abstract

**Objective:**

To investigate the effectiveness and mechanism of the Chinese herbal formula Sini Tang (SNT) which consists of* Aconitum carmichaelii* (Fuzi),* Zingiber officinale* (Gan Jiang), and* Glycyrrhiza uralensis* (Gancao) in heart failure after myocardial infarction in rats.

**Methods:**

We established the heart failure after myocardial infarction in model of SD rats by ligating the anterior descending branch of left coronary artery. Rats were randomly divided into six experimental groups: Sham operation group, HF group, Benazepril group, high dose of SNT group, medium dose of SNT group, and low dose of SNT group. Drugs were administered by oral gavage for eight weeks. The detection indexes include left ventricular function by echocardiogram, Collagen Volume Fraction by Masson staining, level of Plasma Renin, Angiotensin II and Aldosterone by radioimmunoassay, protein and gene level of ACE and AT1R by western-blot, and real-time PCR.

**Results:**

The outcomes of this study indicated that SNT significantly improved the LVEF and LVFS, thickened both LVAWd and LVAWs, and reduced LVIDs in heart failure after myocardial infarction in rats when compared with control group (*P* < 0.05). Besides, SNT significantly reduced the Collagen Volume Fraction (*P* < 0.05). The results of radioimmunoassay showed that SNT decreased the level of Plasma Renin, Angiotensin II, and Aldosterone (*P* < 0.05). The outcomes of western-blot and real-time PCR analysis showed that SNT significantly downregulated the protein and gene level of ACE and AT1R (*P* < 0.05).

**Conclusions:**

The Chinese herbal formula SNT could improve left ventricular systolic function in heart failure after myocardial infarction in rats and decreased the level of Plasma Renin, Angiotensin II, and Aldosterone, as well as downregulating the protein and gene level of ACE and AT1R. Therefore, SNT has potential benefits of improving cardiac function by inhibiting the excessive activation of Renin-Angiotensin-Aldosterone system in heart failure after myocardial infarction in rats.

## 1. Introduction

HF is a clinical syndrome characterized by typical symptoms (e.g., breathlessness, ankle swelling, and fatigue) that may be accompanied by signs (e.g., elevated jugular venous pressure, pulmonary crackles, and peripheral oedema) caused by a structural and/or functional cardiac abnormality, resulting in a reduced cardiac output and/or elevated intracardiac pressures at rest or during stress [1]. According to the reports of World Health Organization (WHO) in 2010, cardiovascular disease is the leading cause of death in humans. What is worse, nearly 290 million patients suffer from cardiovascular disease and 4.5 million patients were diagnosed with heart failure (HF) in China. In Europe, about 26 million people suffer from HF [2].

At present, coronary heart disease has become the primary cause of heart failure and accounts for more than 50% in Europe and North America and 30% to 40% in the East Asia and Latin America [1]. Due to the high morbidity and mortality, HF has become the great burden and challenges for the social economy and healthcare resources. Recently, the treatment of heart failure is mainly based on western medicine in clinical practice [3–6]. However, our traditional Chinese medicine (TCM) also plays an important role in the prevention and treatment of HF [7–13].

In the clinical practice, blood stasis syndrome and Yang deficiency syndrome are both the most common manifestations of heart failure. However, previous studies have focused on the study of blood stasis syndrome. The studies about Yang deficiency syndrome are few. The treatment based on the theory of* warm Yang *has obtained significant clinical efficacy. As the representative decoction of* warm Yang*, Sini Tang (SNT) which originated from* Shang Han Lun* is composed of three kinds of traditional Chinese herbs, namely,* Aconitum carmichaelii* (Fuzi),* Zingiber officinale* (Gan Jiang), and* Glycyrrhiza uralensis* (Gancao). The clinical efficacy of SNT had been confirmed by a large number of clinical studies, while the specific mechanism of its efficacy remains unknown. Therefore, we conducted this trial to investigate the effect of SNT on cardiac function in heart failure after myocardial infarction in rats and explore the specific mechanism of SNT in order to provide a scientific basis for the prevention and treatment of heart failure with SNT.

## 2. Materials and Methods

We used 90 male Sprague-Dawley rats (180–200 g) provided by the Vital Laboratory Animal Technology Company (Beijing, China) for this study. The experimental protocol and the use of animals were approved by the Animal Use and Management Ethics Committee of the Dongzhimen Hospital affiliated to Beijing University of Chinese Medicine. The experimental design and implementation were in accordance with the “Beijing experimental animal regulations” and the United States National Institutes of Health (NIH) “Guide for the Care and Use of Laboratory Animals”. Preoperative and postoperative experimental animals were kept in the Key Laboratory of Chinese Internal Medicine (Beijing University of Chinese Medicine) animal barrier system (temperature within 24–26°C, relative humidity 50% to 60%). All the animals were adapted for seven days before the experiment.

Rats were randomly divided into six experimental groups: Sham operation group (control group with no MI nor HF, treated with deionized water after heart failure, *n* = 15), HF group (Heart Failure group, treated with deionized water after heart failure, *n* = 15), Benazepril group (Benazepril treated after heart failure, *n* = 15), high dose of SNT group (high dose of SNT treated after heart failure, *n* = 15), medium dose of SNT group (medium dose of SNT treated after heart failure, *n* = 15), and low dose of SNT group (low dose of SNT treated after heart failure, *n* = 15).

### 2.1. Preparation for Heart Failure after Myocardial Infarction Model of SD Rats

Sprague-Dawley rats were injected intraperitoneally with pentobarbital sodium (50 mg/kg). Tracheal intubation was connected to an animal ventilator (DH-140, Zhejiang, China). After thoracotomy at the third or fourth intercostal space, the anterior descending branch of left coronary artery was ligated. Once the rats have ventricular fibrillation during the operation, we injected the rats with 1% lidocaine (1 mg/kg), then closed the thorax, and pulled out of the ventilator after spontaneous respiration was restored. All rats were intramuscularly injected with penicillin intramuscularly within three days after surgery. One week after ligation, Electrocardiogram (ECG) was performed, and rats with pathologic *Q* < 6 infarct-related ECG leads were eliminated. Four weeks after ligation, Echocardiography was performed, and rats with left ventricular ejection fraction ≤ 45% were diagnosed as having heart failure [14]. During model establishment, the general state of the animals, including mental state, level of activity, body weight, food and water intake, urine volume and color, stool condition, was recorded every day. Heart failure rats with Yang deficiency syndrome were selected according to the manifestation of animals, such as mental sluggishness, reduction of physical activity, clear urine, loose stool, and cold limbs.

### 2.2. Medication Treatment

When the animal model of heart failure was successful at four weeks after ligation according to the results of Electrocardiogram and Echocardiography, we screened the heart failure rats with Yang deficiency syndrome and divided them into six experimental groups randomly: Sham operation group, Heart Failure group, Benazepril group, high dose of SNT group, and medium dose of and low dose of SNT group. Drugs were administered by oral gavage for eight weeks. The SNT was purchased from Dongzhimen Hospital affiliated to Beijing University of Chinese Medicine. The dosage of the SNT was in accordance with the records of* Shang Han Lun* by Zhang Zhongjing (*Aconitum carmichaelii*: 15 g,* Zingiber officinale*: 20 g,* Glycyrrhiza uralensis*: 30 g). The SNT was administered once daily (11.14 g/kg/d in the high dose group of SNT, 5.57 g/kg/d in the medium dose group of SNT, and 2.79 g/kg/d in the low dose group of SNT) to rats in the SNT group. Benazepril was administered once daily (0.86 mg/kg/d) to rats in the Benazepril group, which was supplied by Beijing Novartis Pharma Ltd. (China).

### 2.3. Measurement of Left Ventricular Function by Echocardiogram

The Left ventricular ejection fraction (LVEF), Left ventricular fractional shortening (LVFS), Left ventricular inner diameters (LVID), and left ventricular anterior wall thickness (LVAWT) were measured at least three consecutive cardiac cycles by echocardiogram (Vevo 770, VisualSonics Inc., USA). The two-dimensional short-axis view of the left ventricular was obtained at the level of the papillary muscle along with the M-mode recordings.

### 2.4. Measurement of Collagen Volume Fraction by Masson Staining of Heart

The hearts were fixed in a 4% paraformaldehyde solution for 48 hours and then embedded in paraffin. The 5 *μ*m thick serial sections cut from paraffin blocks were stained with Masson trichrome solutions. Images of these stained sections were obtained using a light microscope. The Collagen Volume Fraction (CVF) was obtained by using image analysis software (Image-Pro Plus 6.0).

### 2.5. Measurement of Plasma Renin, Angiotensin II, and Aldosterone by Radioimmunoassay

The blood was collected from aorta abdominalis. The Plasma Renin, Angiotensin II, and Aldosterone levels were measured by radioimmunoassay. The test kits were purchased from Beijing Sino-UK institute of Biological Technology (China).

### 2.6. Western-Blot Analysis

Fifty micrograms of cell lysates and myocardial tissue were separated on 10% sodium dodecyl sulfate-polyacrylamide gel electrophoresis (SDS-PAGE) gels and then transferred onto nitrocellulose membranes. Anti-Angiotensin converting enzyme 1 antibody (ACE) (ab216476) and anti-Angiotensin II type 1 receptor antibody (AT1R) (ab18801) were purchased from Abcam, Cambridge, UK. HRP conjugated immunoglobulin was used as a secondary antibody (Jackson ImmunoResearch Laboratories). West Pico Chemiluminescent (Pierce) was used as the substrate to visualize protein bands and quantified using densitometry image analysis software (Image Master VDS; Pharmacia Biotech).

### 2.7. Real-Time PCR Analysis

Total RNA was extracted from the myocardial infarcted marginal area of rats using TRIzol regent (Invitrogen). DNase-treated RNA was used for first strand cDNA synthesis using M-MLV reverse transcriptase (Promega) and oligo (dT) according to the manufacture's protocols and 1 *μ*L cDNA samples were used for conventional PCR amplifications. Real-time quantitative PCR analysis was performed in a real-time PCR system (StepOne, Applied Biosystems) and the expression levels of ACE and AT1R were normalized to GAPDH determined by a SYBR Green-based comparative cycle threshold CT method. Real-time PCR primers sequences are as follows: ACE-F: 5′-GCCATGAAGTTGGGCTACAG-3′, ACE-R: 5′-TCTGTGACGAGCCATTCAGT-3′, AT1R-F: 5′-TACGCCAGTGTGTTCCTTCT-3′, AT1R-R: 5′-ATGATGCAGGTGACTTTGGC-3′, GAPDH-F: 5′-CAACTCCCTCAAGATTGTCAGCAA-3′, GAPDH-R: 5′-GGCATGGACTGTGGTCATGA-3′.

### 2.8. Statistical Methods

We used the software program SPSS 20.0 to conduct the statistical analysis. All results were expressed as mean ± SD. For multiple comparisons each value was compared by one way ANOVA following Dunnett test when each datum conformed to normal distribution, while the non-normally distributed continuous data were compared by using nonparametric tests. A value of *P* < 0.05 was considered statistically significant.

## 3. Results

Five of 15 animals in the HF group, 1 of 15 in the Benazepril group, 2 of 15 in the high dose of SNT group, 2 of 15 in the medium dose of SNT group, and 3 of 15 in the low dose of SNT group died within the eight-week period after coronary artery ligation.

### 3.1. Changes in Left Ventricular Function by Echocardiogram

*LVEF*. Compared with Sham operation group, LVEF was significantly reduced in the HF group. Compared with the HF group, LVEF in Benazepril group, the high dose of SNT group, medium dose of SNT group, and low dose of SNT group were significantly increased. There was no significant difference in LVEF among high dose of SNT group, medium dose group of SNT, and low dose of SNT group.

*LVFS*. Compared with Sham operation group, LVFS was decreased significantly in HF group. Compared with HF group, LVFS in Benazepril group, high dose of SNT group, and middle dose of SNT group were significantly increased. LVFS had no significant difference between HF group and low dose of SNT group. There was no significant difference in LVFS among high dose of SNT group, medium dose of SNT group, and low dose of SNT group.

*LVAWT*. LVAWT includes left ventricular end-diastolic anterior wall thickness (LVAWd) and left ventricular end-systolic anterior wall thickness (LVAWs). Compared with Sham operation group, LVAWd and LVAWs were significantly decreased in HF group. Compared with HF group, LVAWd and LVAWs were significantly increased in Benazepril group, high dose of SNT group, medium dose of SNT group, and low dose of SNT group. There was no significant difference in LVEF among those three kinds of SNT group.

**Figure 1:**
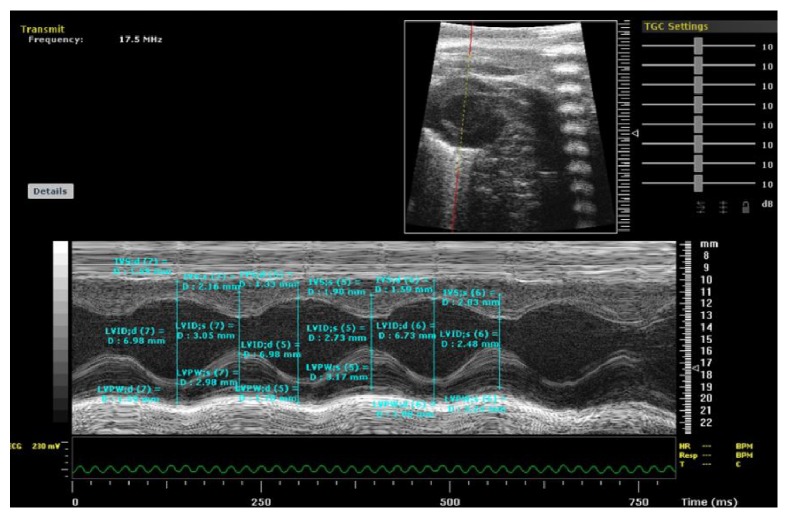
Representative tracing of echocardiography: LV function.

*LVID*. LVID include left ventricular end-diastolic inner diameter (LVIDd) and left ventricular end-systolic inner diameter (LVIDs). Compared with Sham operation group, LVIDs in HF group was increased significantly. Compared with HF group, LVIDs in Benazepril group, high dose of SNT group, and middle dose of SNT group were significantly decreased. LVIDs had no significant difference between HF group and low dose of SNT group. There was no significant difference in LVIDd among Sham operation group, HF group, Benazepril group, and the three kinds of SNT groups (Table 1 and Figure 1).

### 3.2. Changes in Collagen Volume Fraction by Masson Staining

**Figure 2:**
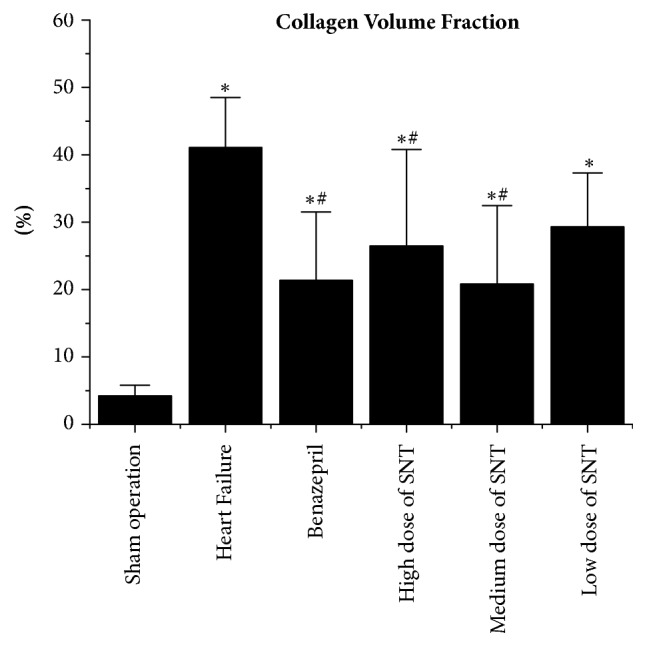
Changes in Collagen Volume Fraction. SNT: Sini Tang; ^*∗*^*P* < 0.05 versus Sham operation group; ^#^*P* < 0.05 versus Heart Failure group.

Compared with Sham operation group, the Collagen Volume Fraction (CVF) significantly increased in HF group. Compared with the HF group, CVF was significantly decreased in Benazepril group, high dose of SNT group, and medium dose of SNT group. CVF had no significant difference between low dose of SNT group and HF group. There was no significant difference among high dose of SNT group, medium dose group of SNT, and low dose of SNT group (Figure 2).

### 3.3. Changes in Plasma Renin, Angiotensin II, and Aldosterone by Radioimmunoassay

**Figure 3:**
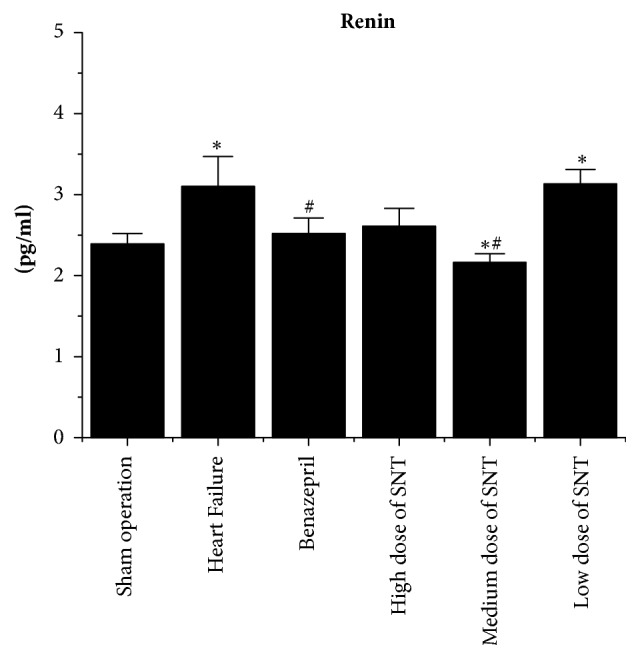
Changes in Plasma Renin by radioimmunoassay. SNT: Sini Tang; ^*∗*^*P* < 0.05 versus Sham operation group; ^#^*P* < 0.05 versus Heart Failure group.

**Figure 4:**
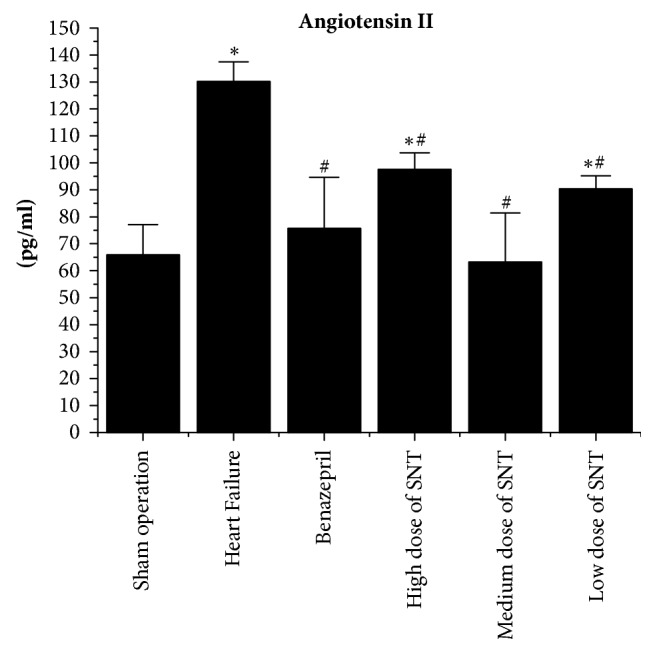
Changes in plasma Angiotensin II by radioimmunoassay. SNT: Sini Tang; ^*∗*^*P* < 0.05 versus Sham operation group; ^#^*P* < 0.05 versus Heart Failure group.

**Figure 5:**
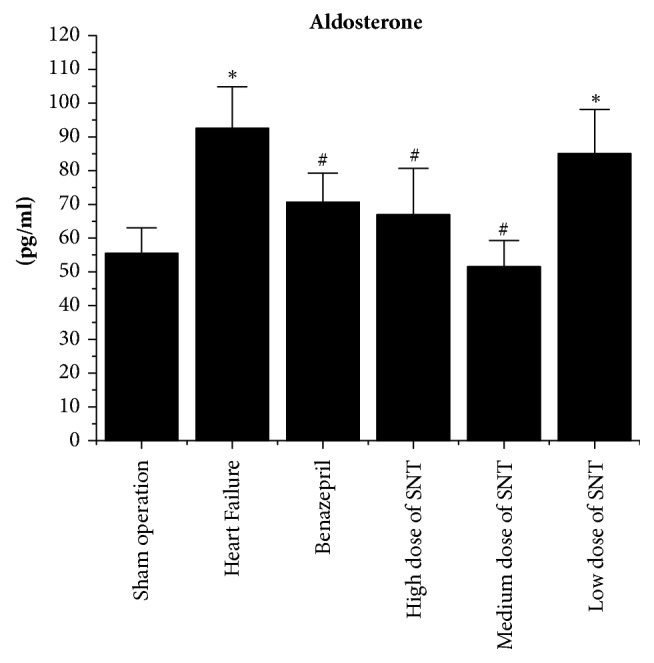
Changes in plasma Aldosterone by radioimmunoassay. SNT: Sini Tang; ^*∗*^*P* < 0.05 versus Sham operation group; ^#^*P* < 0.05 versus Heart Failure group.

Compared with Sham operation group, the level of Plasma Renin, Angiotensin II, and Aldosterone was significantly increased in the HF group. Compared with the HF group, the level of Plasma Renin, Angiotensin II, and Aldosterone was significantly decreased in Benazepril group, the medium dose of SNT group, and low dose of SNT group. The high dose of SNT group significantly reduced the level of Angiotensin II and Aldosterone. The low dose of SNT group only significantly reduced the level of Angiotensin II. There was no significant difference among high dose of SNT group, medium dose group of SNT, and low dose of SNT group (Figures 3–5).

### 3.4. Changes in Protein Expression Level of ACE and ATIR by Western-Blot Analysis

**Figure 6:**
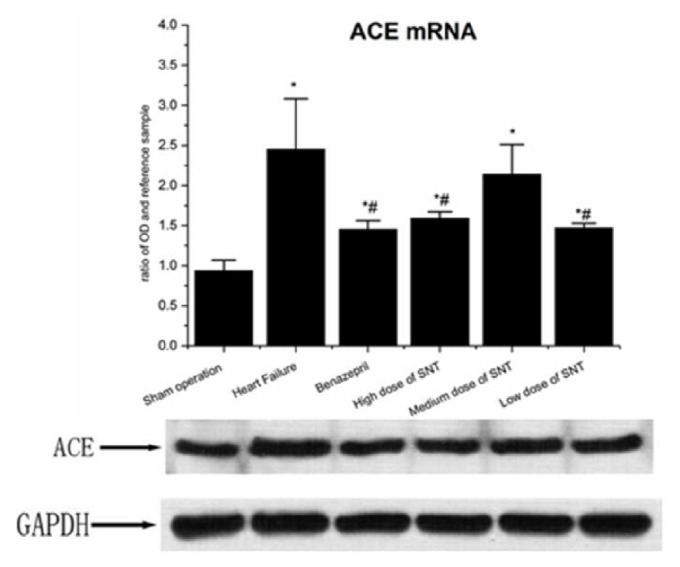
Changes in ACE mRNA and ACE protein. SNT: Sini Tang; ^*∗*^*P* < 0.05 versus Sham operation group; ^#^*P* < 0.05 versus Heart Failure group.

**Figure 7:**
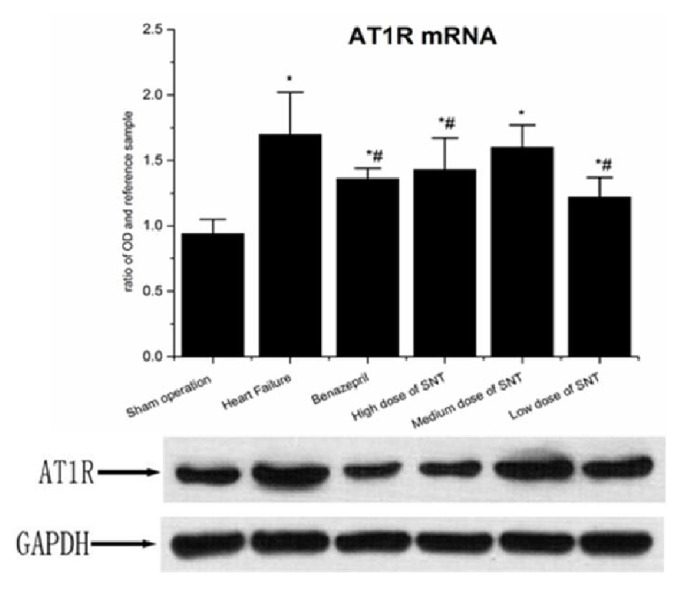
Changes in AT1R mRNA and ATIR protein. SNT: Sini Tang; ^*∗*^*P* < 0.05 versus Sham operation group; ^#^*P* < 0.05 versus Heart Failure group.

When compared with Sham operation group, HF group had significantly upregulated protein expression level of ACE and ATIR. Compared with the HF group, the Benazepril group, high dose of SNT group, and low dose of SNT group had significantly downregulated expression level of ACE and ATIR protein. The medium dose of SNT group only significantly downregulated the expression level of ACE protein. There was no significant difference among high dose of SNT group, medium dose of SNT group, and low dose of SNT group (Figures 6 and 7).

### 3.5. Changes in Gene Expression Level of ACE mRNA and ATIR mRNA by Real-Time PCR Analysis

When compared with Sham operation group, HF group had significantly upregulated gene expression level of ACE mRNA and ATIR mRNA. Compared with the HF group, the Benazepril group, high dose of SNT group, and low dose of SNT group had significantly downregulated gene expression level of ACE mRNA and ATIR mRNA. The gene expression level of ACE mRNA and ATIR mRNA had no significant difference between medium dose of SNT group and HF group. There was no significant difference among high dose of SNT group, medium dose group of SNT, and low dose of SNT group (Figures 6 and 7).

## 4. Discussion

In our study, all the animals were adapted for seven days before the experiment. One week after ligation, Electrocardiogram (ECG) was performed. Four weeks after ligation, Echocardiography was performed. When the animal model of heart failure was successful at four weeks after ligation according to the results of Electrocardiogram and Echocardiography, we screened the model rats and divided them into six experimental groups randomly. Drugs of each group were administered by oral gavage for eight weeks. Eight weeks after treatment, Electrocardiogram and Echocardiography were performed once again in order to evaluate the effectiveness of Sini decoction. The blood and heart tissue of all rats were collected on the same day after Electrocardiogram and Echocardiography.

Previous studies revealed that SNT has the potential to improve early ventricular remodeling after myocardial infarction [15] and a benefit of anti-inflammatory effects in MI rats [16]. The study by Zhou et al. [17] revealed that SNT could protect myocardium in isoproterenol- (ISO-) induced myocardial injury. Liao et al. [18] found that SNT could effectively inhibit ISO-induced myocardial fibrosis. The trial by Zhao et al. found that SNT could provide protection by inhibiting cardiomyocyte apoptosis. Besides, SNT could ease the oxidative stress in Adriamycin- (ADR-) induced heart failure rats [19]. These previous studies confirmed the protection function of SNT in heart failure rats. A former study which was conducted by Liu et al. [15] reported that Chinese medicine formula SNT has the potential to improve early ventricular remodeling and cardiac function after MI. The mechanism may be related to the reduction of the levels of Toll-like receptors (TLR-2 and TLR-4), collagens content and the transformation of growth factor-*β* 1 (TGF-*β* 1) in myocardial tissue. Moreover, a lot of studies have demonstrated that myocardial remodeling and abnormal activation of RAAS are closely correlated. However, the effect of SNT in Renin-Angiotensin-Aldosterone system (RAAS) of heart failure after myocardial infarction in rats remains uncertain.

In this study, SNT has significantly improved the cardiac function in heart failure after myocardial infarction in rats. SNT significantly improved the LVEF and LVFS, thickened both LVAWd and LVAWs, and decreased LVIDs in heart failure after myocardial infarction in rats when compared with control group. The result of Masson scanning showed that SNT significantly reduced the Collagen Volume Fraction which alleviated the condition of collagen deposition. The results of radioimmunoassay indicated that SNT decreased the level of Plasma Renin, Angiotensin II, and Aldosterone. The outcomes of western-blot and real-time PCR analysis showed that SNT significantly downregulated the protein and gene level of ACE and AT1R.

Previous studies had confirmed the clinical efficacy of SNT, and the results of our study revealed that SNT improved the cardiac function in heart failure after myocardial infarction in rats by inhibiting the activation of RAAS. RAAS was considered to be a simple neurohumoral regulation mechanism at first, while after the research of RAAS blocker, such as Renin inhibitors and Angiotensin converting enzyme inhibitors (ACEI), the role of RAAS system has been gradually paid attention to. In the cardiovascular field, pathological activated RAAS can lead to excessive vasoconstriction, hyperplasia of vascular smooth muscle and myocardial hypertrophy, and fibrosis. Inhibiting the RAAS had benefits among patients with hypertension, acute myocardial infarction, and heart failure [20–23]. Therefore, inhibiting excessive activated RAAS system has become an important target for clinical prevention and treatment of heart failure.

## 5. Conclusions

The Chinese herbal formula SNT could improve left ventricular systolic function in heart failure after myocardial infarction in rats and decreased the level of Plasma Renin, Angiotensin II, and Aldosterone, as well as downregulating the protein and gene level of ACE and AT1R. Therefore, SNT has potential benefits of improving cardiac function by inhibiting the excessive activation of Renin-Angiotensin-Aldosterone system in heart failure after myocardial infarction in rats.

## Figures and Tables

**Table 1:** LV function in rats eight weeks post-HF.

Group	*n*	LVEF	LVFS	LVAWd	LVAWs	LVIDd	LVIDs
Sham operation	10	72.97 ± 6.77	44.17 ± 6.35	0.80 ± 0.13	0.98 ± 0.12	8.47 ± 1.23	4.77 ± 1.05
Heart Failure	10	29.38 ± 5.12^*∗*^	14.57 ± 2.71^*∗*^	0.61 ± 0.10^*∗*^	0.72 ± 0.12^*∗*^	9.58 ± 1.44	8.20 ± 1.34^*∗*^
Benazepril	10	52.46 ± 10.59^*∗*#^	28.65 ± 7.11^*∗*#^	0.79 ± 0.11^#^	0.97 ± 0.12^#^	9.17 ± 1.56	6.63 ± 1.62^*∗*#^
High dose of SNT	10	55.22 ± 8.67^*∗*#^	29.94 ± 5.39^*∗*#^	0.85 ± 0.10^#^	0.94 ± 0.12^#^	7.54 ± 2.03^#^	5.36 ± 1.82^#^
Medium dose of SNT	10	53.03 ± 2.75^*∗*#^	28.77 ± 1.80^*∗*#^	0.85 ± 0.13^#^	1.09 ± 0.17^#^	9.59 ± 1.50	6.83 ± 1.13^*∗*#^
Low dose of SNT	10	43.80 ± 5.65^*∗*#^	23.09 ± 3.48^*∗*^	0.84 ± 0.06^#^	1.06 ± 0.09^#^	10.62 ± 1.38^*∗*^	8.16 ± 1.13^*∗*^

LVEF: left ventricular ejection fraction; LVFS: left ventricular fractional shortening; LVAWd: left ventricular end-diastolic anterior wall thickness; LVAWs: left ventricular end-systolic anterior wall thickness; LVIDd: left ventricular end-diastolic inner diameter; LVIDs: left ventricular end-systolic inner diameter; ^*∗*^*P* < 0.05 versus Sham operation group; ^#^*P* < 0.05 versus Heart Failure group.

## Data Availability

The data used to support the findings of this study are available from the corresponding author upon request.
